# An Infrared Ultra-Broadband Absorber Based on MIM Structure

**DOI:** 10.3390/nano12193477

**Published:** 2022-10-04

**Authors:** Meichen Li, Guan Wang, Yang Gao, Yachen Gao

**Affiliations:** Electronic Engineering College, Heilongjiang University, Harbin 150080, China

**Keywords:** metamaterial, infrared, ultra-broadband absorption, local surface plasmon resonance

## Abstract

We designed an infrared ultra-broadband metal–insulator–metal (MIM)-based absorber which is composed of a top layer with four different chromium (Cr) nano-rings, an intermediate media of aluminum trioxide (Al_2_O_3_), and a bottom layer of tungsten (W). By using the finite-difference time-domain (FDTD), the absorption performance of the absorber was studied theoretically. The results indicate that the average absorption of the absorber can reach 94.84% in the wavelength range of 800–3000 nm. The analysis of the electric and magnetic field indicates that the ultra-broadband absorption rate results from the effect of local surface plasmon resonance (LSPR). After that, the effect of structural parameters, metal and dielectric materials on the absorptivity of the absorber was also discussed. Finally, the effect of incidence angle on absorption was investigated. It was found that it is not sensitive to incidence angle; even when incidence angle is 30°, average absorptivity can reach 90%. The absorber is easy to manufacture and simple in structure, and can be applied in infrared detection and optical imaging.

## 1. Introduction

Metamaterials (MMs) are artificial materials consisting of subwavelength periodic arrays that have received widespread attention because they possess properties not found in natural materials [1,2]. In 2008, Landy et al. first proposed a narrow-band perfect metamaterial absorber, which is based on metal–insulator–metal (MIM) [3], demonstrating that metamaterials can effectively absorb electromagnetic waves. Since then, the operating region of the absorber has been extended from the microwave band to the visible [4], infrared [5,6,7,8] and terahertz bands [9,10]. At the same time, various single-band [11], dual-band [12], multi-band [13] broadband absorbers [14] have also been hot topics of research. Especially, broadband absorbers have been extensively studied due to their potential application in photovoltaic devices [15,16,17], communication [18,19], photoelectric detection [20,21,22,23], solar Cell [24,25,26] and other areas [27]. In 2017, Dong Wu et al. proposed a solar absorber consisting of tungsten spheres embedded in SiO_2_. In the wavelength range of 435–1520 nm, the absorber can reach an average absorptivity of 99% and it is polarization-independent [28]. In 2018, Dewang Huo et al. designed an absorber using TiN. From 400 to 1500 nm, the absorber can attain an average of 99.6% absorption [29]. In 2019, Wu Biao et al. proposed a Ti-Si-Ti trilayer-based structure with an absorption bandwidth of 1376 nm and a spectral average absorbance higher than 94% in the visible to near-infrared band, and the absorber is polarization-independent and angle-insensitive [30]. In 2020, Hailiang Li et al. used a cross-shaped structure composed of refractory metals Ti, TiN and SiO_2_ to make a structure with an absorption bandwidth of 1182 nm, and the absorber can absorb most of the solar energy [31]. In 2021, Shengxi Jiao et al. proposed a absorber consisting of Ti-Al_2_O_3_-W. From 500 to 1800 nm, the absorber has an average absorption of 94% [32]. From researchers’ studies above, we can find that metal nanostructures can broaden the absorption spectra. However, the absorption bandwidth of these absorbers is still limited, and it is necessary to broaden the bandwidth of the absorber further.

In this paper, we proposed an ultra-broadband absorber composed of nano-rings which is based on MIM structure, and studied theoretically the absorption properties of the absorber by using the finite-difference time-domain (FDTD) method. The absorption of the absorber can reach 94.84% in the wavelength range of 800–3000 nm. The proposed absorber has a wider absorption bandwidth, insensitive to incidence angle, has a simple structure and the manufacturing process of the structure is simple.

## 2. Structure and the Simulation Methods

**Figure 1:**
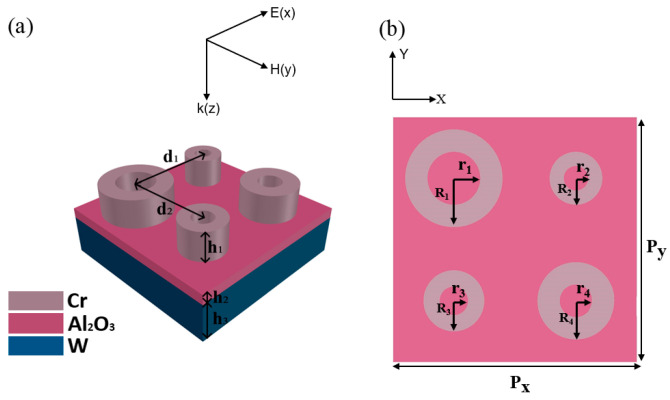
(**a**) Structure diagram of one unit of absorber; (**b**) Top view of unit structure.

Previous research has indicated that nano-rings can achieve broadband absorption, and the average absorptivity can reach more than 90% [33]. So, in this paper, we designed a new structure based on nano-rings to realize a much broader absorption range. The structure of the proposed absorber is shown in Figure 1. One cell of the absorber is shown in Figure 1a, comprising a top layer with four Cr nano-rings, a dielectric layer of Al_2_O_3_ and a bottom layer of W. The top view of the unit structure is shown in Figure 1b. The four nano-rings are named ring 1, ring 2, ring 3 and ring 4. The structural parameters are as follows: the thickness of the Cr nano-rings h_1_ = 200 nm, the thickness of Al_2_O_3_ h_2_ = 70 nm, the thickness of W h_3_ = 200 nm, the inner radius r_1_ = 130 nm and the outer radius R_1_ = 240 nm for ring 1, the inner radius r_2_ = 60 nm and the outer radius R_2_ = 130 nm for ring_2_, inner radius r_3_ = 70 nm and R_3_ = 150 nm for ring 3, the inner radius r_4_ = 80 nm and outer radius R_4_ = 190 nm for ring 4, the distance between ring 1 and ring 2 d_1_ = 600 nm, the distance between ring 1 and ring 3 d_2_ = 600 nm, the space period p = 1200 nm, and the material properties of the above are referred to from Palik [34].

The absorption properties, electric and magnetic fields distribution of the absorber are analyzed by using FDTD solution. The plane wave is incident vertically along the *z*-axis direction [35]. The *x* and *y* directions are set as periodic boundary conditions. The *z* direction is set as perfect matched layer (PML). Under oblique incidence, we choose the Broadband Fixed Angle Source Technique (BFAST) mode. The absorption of the absorber can be calculated by using the Equation (1) [36]:

(1) A=1−T(ω)−R(ω)

where T(ω) represents transmission and R(ω) represents reflection, and $R\left(\omega \right)={\left|S_{11}\left(\omega \right)\right|}^{2}$, $T\left(\omega \right)={\left|S_{21}\left(\omega \right)\right|}^{2}$, where $S_{11}$ and $S_{21}$ are the reflection and transmission coefficients of the absorber, respectively. Since the thickness of W is 200 nm, which is sufficient to block all transmissions in the operating wavelength range, the formula can be simplified as $A=1-R\left(\omega \right)=1-{\left|S_{11}\left(\omega \right)\right|}^{2}\;$ [37].

## 3. Results and Discussion

**Figure 2:**
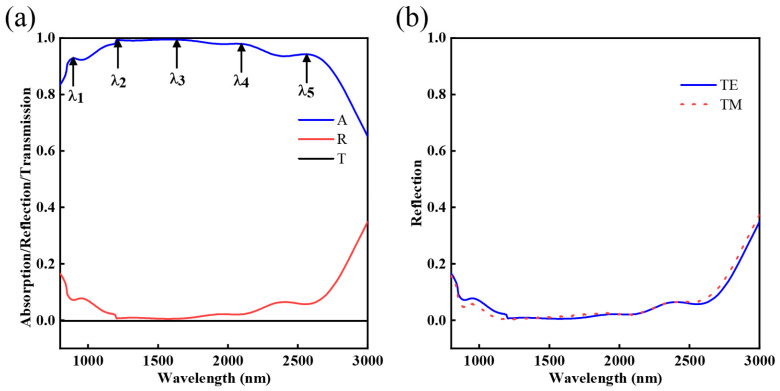
(**a**) Simulated absorption, reflection and transmission spectra of the broadband; (**b**) Reflection spectra in TE and TM polarization modes.

The reflection spectrum, absorption spectrum and transmission spectrum of the absorber are displayed in Figure 2a. The average absorptivity of the absorber is 94.84% from 800 to 3000 nm. There are five resonance peaks that can be seen from the spectra, which are λ_1_ = 886 nm, λ_2_ = 1204 nm, λ_3_ = 1561 nm, λ_4_ = 2054 nm and λ_5_ = 2563 nm, with absorption rates of 92.80%, 99.37%, 99.44%, 98.01% and 94.32%, respectively. The reflection spectra of TE and TM polarization modes at normal incidence are shown in Figure 2b, where two reflection spectra do not overlap, which is due to the fact that the absorber structural unit is not completely symmetric. Moreover, in the wavelength range of 800–3000 nm, the average absorptivity of the absorber for both TM and TE polarization states can reach 94%, achieving broadband absorption in both polarization modes.

**Figure 3:**
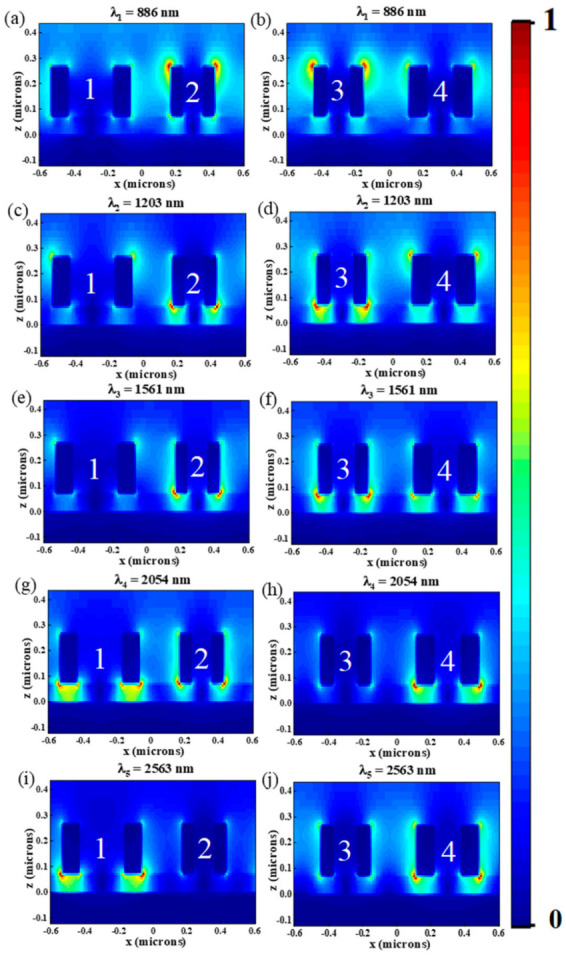
In the *x*–*z* plane, the electric field distribution of the absorber at (**a**,**b**) λ_1_ = 886 nm, (**c**,**d**) λ_2_ = 1204 nm, (**e**,**f**) λ_3_ = 1561 nm, (**g**,**h**) λ_4_ = 2054 nm, (**i**,**j**) λ_5_ = 2563 nm.

**Figure 4:**
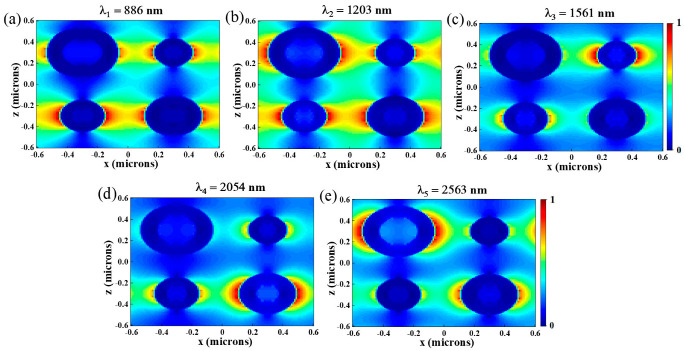
In the *x*–*y* plane, the electric field distribution of the absorber at (**a**) λ_1_ = 886 nm, (**b**) λ_2_ = 1204 nm, (**c**) λ_3_ = 1561 nm, (**d**) λ_4_ = 2054 nm, (**e**) λ_5_ = 2563 nm.

In order to understand the physical mechanism of the ultra-broadband absorption, at those five resonance peaks (λ_1_ = 886 nm, λ_2_ = 1204 nm, λ_3_ = 1561 nm, λ_4_ = 2054 nm and λ_5_ = 2563 nm), the electric field distributions in the *x*–*z* and *x*–*y* planes are calculated and given in Figure 3 and Figure 4. As shown in Figure 3a,b at λ = 886 nm, the enhanced electric field is mainly distributed at the interface of ring 2, ring 3, ring 4 and air, which indicates that strong LSPR is generated, and abundant electrons concentrated surrounding the Cr nano-rings, strengthening the electric field [38]. The principle of LSPR generation is when the light incident on the nanostructures is composed of noble metals, if the incident photon frequency suited the overall vibration frequency of metal nanostructures, the nanostructures will have a strong absorption effect on the photon energy, and LSPR will occur [39]. We can see from Figure 4a that there is a dipole resonance between ring 1 and ring 2 (ring 3 and ring 4). When λ = 1203 nm, the electric field distributions of the absorber are shown in Figure 3c,d and Figure 4b. It is obvious that the enhanced electric field is mainly distributed at the interface of ring 1, ring 4 and air, as well as ring 2, ring 3 and Al_2_O_3_. As shown in Figure 4b, we can also see the dipole resonance occurs between ring 1 and ring 2 (ring 3 and ring 4). While at the wavelengths of 1491 nm, 2054 nm and 2563 nm, as shown in Figure 3e–j and Figure 4c–e, LSPR is mainly distributed between the nano-rings and the intermediate dielectric layer.

**Figure 5:**
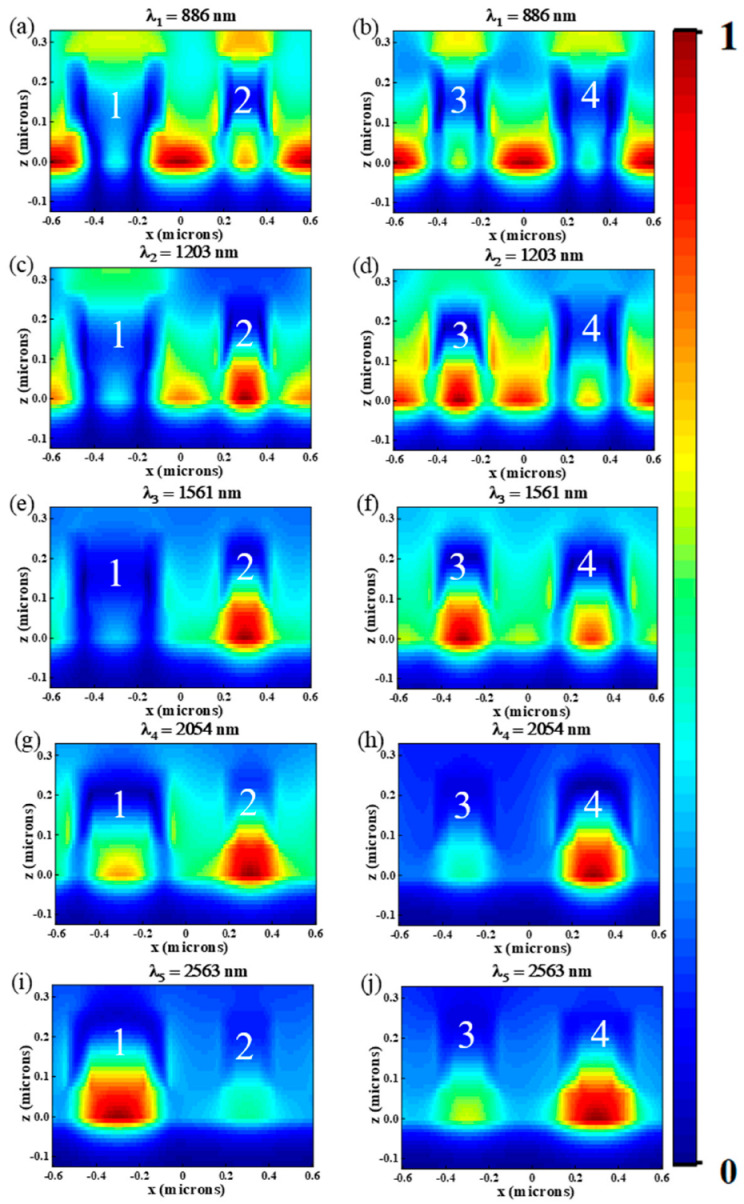
In the *x*–*y* plane, the magnetic field distribution of the absorber at (**a**,**b**) λ_1_ = 886 nm, (**c**,**d**) λ_2_ = 1204 nm, (**e**,**f**) λ_3_ = 1561 nm, (**g**,**h**) λ_4_ = 2054 nm, (**i**,**j**) λ_5_ = 2563 nm.

Next, in order to further analyze the physical mechanisms of the absorber, we plotted the magnetic distributions in the *x*–*z* plane at these five resonances peaks in Figure 5. As shown in Figure 5a,b, the magnetic field at λ = 886 nm is concentrated in the dielectric layer below the adjacent rings, indicating that LSPR is excited, and in Figure 5c,d, the magnetic field at λ = 1204 nm is concentrated under ring 2 and ring 3, and the magnetic also distributes below the adjacent rings, which indicates that ring 2, ring 3, ring 4 excite LSPR. As shown in Figure 5e,f, at λ = 1561 nm, the magnetic field is mainly distributed under ring 2, ring 3, and ring 4. Compared with Figure 5a,b, the magnetic field concentrated in the dielectric layer below the adjacent rings is weakened, however, the magnetic field under ring 2, ring 3, and ring 4 is enhanced, indicating two different ways of resonance. As shown in Figure 5g,h, the magnetic field at λ = 2054 nm is mainly distributed under ring 2 and ring 4, and a small portion of the magnetic field is distributed under ring 1. Compared with Figure 5c,d, the magnetic field under ring 2, ring 4 is significantly enhanced. In Figure 5i,j, at λ = 2563 nm, the magnetic field is mainly concentrated below ring 1 and ring 4. Compared with Figure 5e–h, the magnetic field under nano-rings are significantly enhanced. By the above analysis, the magnetic field distributions of these five resonance peaks are different, indicating that every resonance peak has a different resonance way.

In addition to the physical mechanism of the ultra-broadband absorption, the influence of structural parameters on the absorption properties of broadband absorber is also studied. Specifically, we studied how the absorption spectrum changes with the thickness of the Cr nano-rings h_1_, the thickness of Al_2_O_3_ h_2_, the distance between ring 1 and ring 2 d_1_ and the distance between ring 1 and ring 3 d_2_.

**Figure 6:**
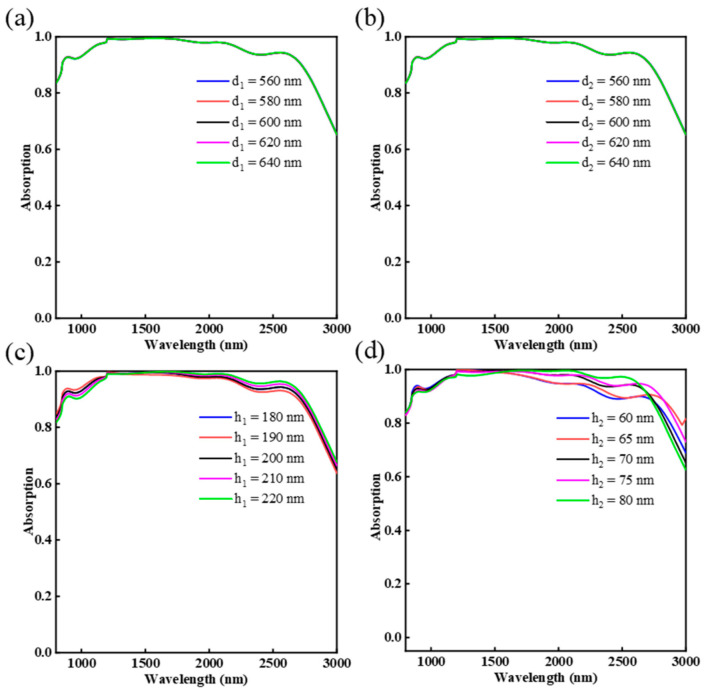
The influence of different structural parameters on the absorption properties: (**a**) distance between ring 1 and ring 2 d_1_; (**b**) distance between ring 1 and ring 3 d_2_; (**c**) thickness of the Cr nano-rings h_1_; (**d**) thickness of Al_2_O_3_ h_2_.

Figure 6a shows the effect of the distance d_1_ between ring 1 and ring 2 (ring 3 and ring 4) on the absorption spectrum. It is obvious that the absorption spectra almost overlap as d_1_ changes from 560 nm to 640 nm with a step of 20 nm. Similarly, Figure 6b shows the effect of different distances d_2_ between ring 1 and ring 3 (ring 2 and ring 4) on the absorption properties, which has the same trend as that in Figure 6a. This is due to the fact that the distance between adjacent rings is large, which leads to weak coupling of plasmon resonance, so the spectral shift is not obvious with the change of d_1_ and d_2_. Next, we demonstrated the effect of h_1_, h_2_ on the absorption properties of the absorber. When h_1_ increases from 180 nm to 220 nm in steps of 10 nm, the absorption spectrum is shown in Figure 6c. From the figure, we can see the average absorptivity of the absorber does not change much. In other words, h_1_ has little influence on the absorption properties. Figure 6d illustrates the absorption spectra whereas h_2_ increases from 60 to 80 nm. It can be found that the average absorption rate of the absorber increases first, reaches the maximum for the absorber when h_2_ = 70 nm, and then decreases. By optimizing the parameters, the average absorption rate of the absorber can be maximized, when d_1_ = 600 nm, d_2_ = 600 nm, h_1_ = 200 nm, h_2_ = 70 nm.

**Figure 7:**
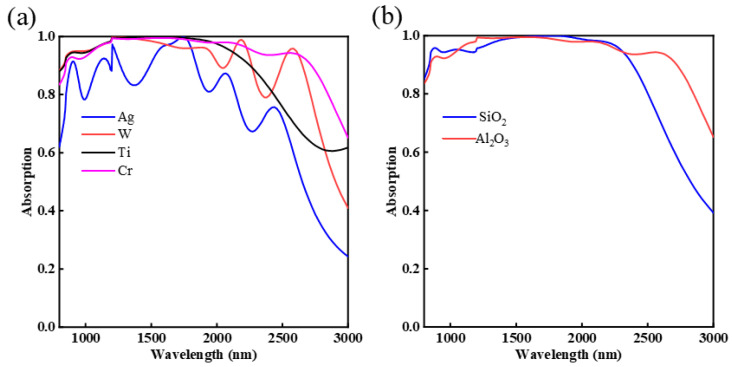
The influence of different materials on the absorption spectrum: (**a**) top metal materials; (**b**) dielectric layer.

As different metal materials’ plasma and collision frequencies are different, the absorption performance of the absorber will also different. Therefore, we selected four metal materials, respectively, silver (Ag), tungsten (W), titanium (Ti) and chromium (Cr), to study absorption performance of the absorber. Figure 7a shows the absorption spectra with these metal materials. From 800 to 3000 nm, we can clearly see that the average absorption rate of the absorber is very low when the top metal is Ag, W and Ti, and the resonance bands of Ag and W are single, which cannot achieve continuous broadband absorption. When the top metal is Ti, the absorber has a broader bandwidth, but it cannot achieve continuous high absorption (above 90%) in the work wavelength of the absorber. However, compared with other metals, when the top layer was chosen as Cr, the average absorption rate can reach the highest. In addition, the refractive index imaginary part of Cr is large, which makes Cr show strong light absorption [40], and it also has a higher melting point and lower price which makes it the most optimal top metal for the absorber. Next, we analyzed the influence of the different intermediate media layer on the absorber. Figure 7b shows the absorption spectra versus SiO_2_ and Al_2_O_3_. The absorption spectrum for Al_2_O_3_ shows wider absorption bandwidth and higher average absorption rate in the working wavelength than SiO_2_. This demonstrates that the dielectric layer with different refractive index (n) can influence the optical properties of the absorber. In this study, Al_2_O_3_ is recognized as the dielectric material for its higher average absorption rate.

**Figure 8:**
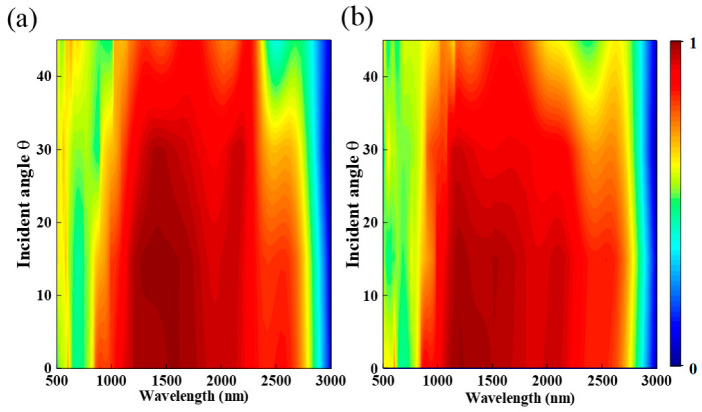
Absorption spectra at different incidence angles under (**a**) TE and (**b**) TM polarizations.

In addition, an ideal broadband absorber should be able to operate at a wide range of oblique incidence angles. Therefore, we studied the effect of different oblique incidence angle on the absorption performance of the absorber. We can see from Figure 8a,b, when the oblique incidence angles increase from 0° to 30° under the TE and TM polarization, from 800 to 3000 nm, the average absorption rate of the absorber can still reach more than 90%, which indicates the absorber is insensitive to incident angle.

For comparison, the absorption properties of our design and reported similar absorber are list in Table 1. It can be found that, compared with the other absorber, the proposed absorber has wider absorption band.

In order to facilitate the production of the proposed absorber later. The proposed preparation method is as follows: the dielectric layer of Al_2_O_3_ can be formed on W substrate by thermal evaporation. Next, nano-rings masks with the same structure as proposed are made, placed on a 200 nm thick photoresist, etched with standard photolithography, then coated with Cr by thermal evaporation, and finally, the excess photoresist rinsed off [43].

## 4. Conclusions

In summary, we designed an ultra-broadband absorber composed of Cr, Al_2_O_3_ and W. The results show that the absorber has an average absorptivity of 94.84% in the wavelength range of 800–3000 nm, and the ultra-broadband absorption originates from LSPR. Moreover, the absorber is insensitive to incident angle. When the incident angle reaches 30°, the absorptivity is still more than 90%. The proposed absorber has a wider absorption bandwidth and great prospects for applications in infrared detection and optical imaging.

## Figures and Tables

**Table 1:** Comparisons of the designed absorber with previous absorbers.

References	Materials Used	Pattern	Absorption Band (>90%) (nm)
[28]	TiN, Al_2_O_3_	Cone	400–1500 (1100)
[29]	Ti, SiO_2_	Elliptical	456–1832 (1376)
[41]	Ti, SiO_2_, Au	Circular	900–1825 (925)
[32]	W, Al_2_O_3_, Ti	Elliptical	500–1800 (1300)
[42]	TiN, TiO_2_	Circular	316–1426 (1110)
proposed	Cr, Al_2_O_3_, W	Ring	800–3000 (2200)

## Data Availability

All content and data have been displayed in the manuscript.

## References

[1] Pendry J.B., Holden A.J., Stewart W.J., Youngs I. (1996). Extremely low frequency plasmons in metallic mesostructures. Phys. Rev. Lett..

[2] Cerjan B., Gerislioglu B., Link S., Nordlander P., Halas N.J., Griep M. (2022). Towards scalable plasmonic Fano-resonant metasurfaces for colorimetric sensing. Nanotechnology.

[3] Landy N.I., Sajuyigbe S., Mock J.J., Smith D.R., Padilla W.J. (2008). Perfect metamaterial absorber. Phys. Rev. Lett..

[4] Wang R., Yue S., Zhang Z., Hou Y., Zhao H., Qu S., Li M., Zhang Z. (2022). Broadband Perfect Absorber in the Visible Range Based on Metasurface Composite Structures. Materials.

[5] Feng Q., Pu M., Hu C., Luo X. (2012). Engineering the dispersion of metamaterial surface for broadband infrared absorption. Opt. Lett..

[6] Xiong Y., Liu X., Zhang J., Wang X., Wang X., Gao J., Yang H. (2022). High-Performance Ultra-Broadband Absorber for Polarized Long-Wavelength Infrared Light Trapping. Coatings.

[7] Deng G., Sun H., Lv K., Yang J., Yin Z., Li Y., Chi B. (2021). Enhanced broadband absorption with a twisted multilayer metal-dielectric stacking metamaterial. Nanoscale Adv..

[8] Lee D., Go M., Kim M., Jang J., Choi C., Kim J.K., Rho J. (2021). Multiple-patterning colloidal lithography-implemented scalable manufacturing of heat-tolerant titanium nitride broadband absorbers in the visible to near-infrared. Microsyst. Nanoeng..

[9] Huang M., Wei K., Wu P., Xu D., Xu Y. (2021). Terahertz Broadband Absorber Based on a Combined Circular Disc Structure. Micromachines.

[10] Luo X., Xiang P., Yu H., Huang S., Yu T., Zhu Y.-F. (2022). Terahertz Metamaterials Broadband Perfect Absorber Based on Molybdenum Disulfide. IEEE Photonics Technol. Lett..

[11] Tao H., Bingham C.M., Strikwerda A.C., Pilon D., Shrekenhamer D., Landy N.I., Fan K., Zhang X., Padilla W.J., Averitt R.D. (2008). Highly flexible wide angle of incidence terahertz metamaterial absorber: Design, fabrication, and characterization. Phys. Rev. B..

[12] Chen K., Adato R., Altug H. (2012). Dual-Band Perfect Absorber for Multispectral Plasmon-Enhanced Infrared Spectroscopy. ACS Nano.

[13] Shen X., Cui T.J., Zhao J., Ma H.F., Jiang W.X., Li H. (2011). Polarization-independent wide-angle triple-band metamaterial absorber. Opt. Express.

[14] Xu K.-D., Li J., Zhang A., Chen Q. (2020). Tunable multi-band terahertz absorber using a single-layer square graphene ring structure with T-shaped graphene strips. Opt. Express.

[15] Ko H., Ko D.-H., Cho Y., Han I.K. (2014). Broadband light absorption using a multilayered gap surface plasmon resonator. Appl. Phys. A-Mater..

[16] Wang J., Zhang W., Zhu M., Yi K., Shao J. (2015). Broadband Perfect Absorber with Titanium Nitride Nano-disk Array. Plasmonics.

[17] Wang J., Zhu M., Sun J., Yi K., Shao J. (2016). A broadband polarization-independent perfect absorber with tapered cylinder structures. Opt. Mater..

[18] Luo H., Cheng Y.Z. (2017). Design of an ultrabroadband visible metamaterial absorber based on three-dimensional metallic nanostructures. Mod. Phys. Lett. B.

[19] Ma L., Xu H., Lu Z., Tan J. (2022). Optically Transparent Broadband Microwave Absorber by Graphene and Metallic Rings. ACS Appl. Mater. Interfaces.

[20] Qi B., Zhao Y., Niu T., Mei Z. (2019). Ultra-broadband metamaterial absorber based on all-metal nanostructures. J. Phys. D Appl. Phys..

[21] Tang J., Xiao Z., Xu K. (2017). Broadband Ultrathin Absorber and Sensing Application Based on Hybrid Materials in Infrared Region. Plasmonics.

[22] Wang B.-X., Wang L.-L., Wang G.-Z., Huang W.-Q., Zhai X., Li X.-F. (2014). Tunable bandwidth of the terahertz metamaterial absorber. Opt. Commun..

[23] Gerislioglu B., Ahmadivand A., Adam J. (2019). Infrared plasmonic photodetectors: The emergence of high photon yield toroidal metadevices. Mater. Today. Chem..

[24] Liu F., Qi L. (2021). A simple two-layer broadband metamaterial absorber for solar cells. Mod. Phys. Lett. B.

[25] Patel S.K., Charola S., Parmar J., Ladumor M. (2019). Broadband metasurface solar absorber in the visible and near-infrared region. Mater. Res. Express.

[26] Zhu L., Jin Y., Liu H., Liu Y. (2020). Ultra-Broadband Absorber Based on Metal-Insulator-Metal Four-Headed Arrow Nanostructure. Plasmonics.

[27] Zhou L., Tan Y., Ji D., Zhu B., Zhang P., Xu J., Gan Q., Yu Z., Zhu J. (2016). Self-assembly of highly efficient, broadband plasmonic absorbers for solar steam generation. Sci. Adv..

[28] Wu D., Liu Y., Xu Z., Yu Z., Yu L., Chen L., Liu C., Li R., Ma R., Zhang J. (2017). Numerical Study of the Wide-angle Polarization-Independent Ultra-Broadband Efficient Selective Solar Absorber in the Entire Solar Spectrum. Sol. RRL.

[29] Huo D., Zhang J., Wang Y., Wang C., Su H., Zhao H. (2018). Broadband Perfect Absorber Based on TiN-Nanocone Metasurface. Nanomaterials.

[30] Wu B., Liu Z., Liu G., Liu X., Tang P., Du G., Yuan W., Liu M. (2019). An ultra-broadband, polarization and angle-insensitive metamaterial light absorber. J. Phys. D Appl. Phys..

[31] Li H., Niu J., Zhang C., Niu G., Ye X., Xie C. (2020). Ultra-Broadband High-Efficiency Solar Absorber Based on Double-Size Cross-Shaped Refractory Metals. Nanomaterials.

[32] Jiao S., Li Y., Yang H., Xu S. (2021). Numerical study of ultra-broadband wide-angle absorber. Results Phys..

[33] Zhou F., Qin F., Yi Z., Yao W., Liu Z., Wu X., Wu P. (2021). Ultra-wideband and wide-angle perfect solar energy absorber based on Ti nanorings surface plasmon resonance. Phys. Chem. Chem. Phys..

[34] Palik E.D. (1998). Handbook of Optical Constants of Solids.

[35] Zhang N., Zhou P., Zhang L., Weng X., Xie J., Deng L. (2015). Ultra-broadband absorption in mid-infrared spectrum with graded permittivity metamaterial waveguide structure. Appl. Phys. B.

[36] Wang J., Lang T., Shen T., Shen C., Hong Z., Lu C. (2020). Numerical Study of an Ultra-Broadband All-Silicon Terahertz Absorber. Appl. Sci..

[37] Deng H., Stan L., Czaplewski D.A., Gao J., Yang X. (2017). Broadband infrared absorbers with stacked double chromium ring resonators. Opt. Express.

[38] Cen C., Zhang Y., Chen X., Yang H., Yi Z., Yao W., Tang Y., Yi Y., Wang J., Wu P. (2020). A dual-band metamaterial absorber for graphene surface plasmon resonance at terahertz frequency. Physica E.

[39] Raether H. (1988). Surface Plasmons on Smooth Surfaces.

[40] Li J., Gan R., Guo Q., Liu H., Xu J., Yi F. (2018). Tailoring optical responses of infrared plasmonic metamaterial absorbers by optical phonons. Opt. Express.

[41] Ding F., Dai J., Chen Y., Zhu J., Jin Y., Bozhevolnyi S.I. (2016). Broadband near-infrared metamaterial absorbers utilizing highly lossy metals. Sci. Rep. UK.

[42] Liu Z., Liu G., Huang Z., Liu X., Fu G. (2018). Ultra-broadband perfect solar absorber by an ultra-thin refractory titanium nitride meta-surface. Sol. Energy Mat. Sol. C.

[43] Zheng Z., Zheng Y., Luo Y., Yi Z., Zhang J., Liu Z., Yang W., Yu Y., Wu X., Wu P. (2022). A switchable terahertz device combining ultra-wideband absorption and ultra-wideband complete reflection. Phys. Chem. Chem. Phys..

